# High-throughput screening of stable and efficient double inorganic halide perovskite materials by DFT

**DOI:** 10.1038/s41598-022-16221-3

**Published:** 2022-07-25

**Authors:** Xinfeng Diao, Yongxin Diao, Yanlin Tang, Gangling Zhao, Qinzhong Gu, Yu Xie, Yebai Shi, Ping Zhu, Liang Zhang

**Affiliations:** 1 College of Electronic and Electrical Engineering, Shangqiu Normal University, Shangqiu, 476000 China; 2 College of Physics, Guizhou University, Guiyang, 550025 China; 3 Henan Zhongfen Instrument Co., Ltd, Shangqiu, 476000 China

**Keywords:** Materials science, Optics and photonics

## Abstract

Perovskite solar cells have become the most promising third-generation solar cells because of their superior physical–chemical properties and high photoelectric conversion efficiency. However, the current obstacles to commercialization of perovskite solar cells are their poor stability and harmful elements. How to find high-efficiency, high-stability and non-toxic perovskite materials from thousands of possible perovskite crystals is the key to solve this problem. In this paper, the inorganic halide double perovskite A_2_BX_6_ and its crystal structure are considered, and the data mining algorithm in informatics is introduced into the high-throughput computing data to analyze various elements in nature to study the perovskite materials that can meet the requirements of high performance. The photoelectric conversion properties and stability of 42 inorganic double perovskite materials are studied based on density functional theory (DFT). The results show that the tolerance factors of 39 crystals are between 0.8 and 1.10, indicating that these crystals have stable perovskite structure. In addition, the dielectric function, PDOS, elastic modulus, shear modulus and poison’s ratio of these crystals are analyzed. According to the above theoretical simulation results, three candidate materials for ideal light absorption are presented. This can provide a theoretical basis for the industrial application of perovskite solar cells.

## Introduction

Halide perovskite materials have shown great potential as light absorbers in perovskite solar cells and which have been aroused great enthusiasm by researchers^1–4^. Because they have many excellent properties such as tunable band gap^5–7^, strong light absorption^8,9^, long carrier diffusion length^10,11^, and small exciton binding energy^12,13^. Since the first application in 2009 for perovskite materials, it has developed rapidly in just a few years^14–18^.The photoelectric conversion efficiency (PCE) of metal halide perovskite solar cells rose from 3.8% for dye-sensitized solar cell configurations to 25.7% for planar heterojunction cells which is certified by National Renewable Energy Laboratory (NREL)^19–22^. However, the current obstacle to commercialization is the stability of perovskite solar cells and its harmful elements^23–26^. The specific solution is to improve humidity stability, allowing modules to operate in outdoor environments without the need for expensive packaging, as well as replacing the harmful element lead (Pb) with other non-toxic metal halides.

At present, these problems have not been well resolved. How to construct perovskite crystal materials that meet the above requirements is the key to solving the problem. As we know that there are 118 elements in the periodic table. It is difficult to screen out high-efficiency, high-stability and non-toxic perovskite materials from thousands of possible combinations for perovskite crystals. Fortunately, the situation would improve if we expand our search from ternary A^+^B^2+^X_3_ perovskites to quaternary A_2_^+^B^4+^X_6_ double perovskites. The combined advantages of the halide double perovskite Cs_2_TiX_6_ with low cost, high efficiency, nontoxicity, stability, and tunable bandgap have also been recently reported^27–32^. On the other hand, many studies have shown that replacing MA^+^ or FA^+^ with cesium ion (Cs^+^) or rubidium ion (Rb^+^) can significantly improve the thermal stability of materials^13^. These perovskite materials are considered suitable as carrier transport materials (CTM) for CsPbBr_3_ and Cs_2_TiBr_6_ in light-collecting or light-emitting devices. Most of double inorganic halide perovskite materials are direct band gap semiconductor materials, and they have strong visible light absorption^33^. There are many tetravalent ions in nature, such as Ge^4+^, Zr^4+^, Sn^4+^, Hf^4+^, Se^4+^, Te^4+^ and Pd^4+^, which can combine with monovalent metal ions Cs^+^ and Rb^+^ to form double perovskite structures. These materials have suitable electronic configurations and are very stable in air exposure.

Herein, the properties of inorganic metal halide perovskite materials are comprehensively discussed based on DFT. And the organization of this paper mainly includes four parts. First of all, we describe the calculation method and specific parameters used in this work. Then, we consider inorganic halide double perovskite A_2_BX_6_ and its crystal structure with the method of high-throughput screening. Thirdly, we further simulation calculated the parameters of these materials, such as band structure (the position of conduction band and valence band), electronic density of states, absorption spectrum, etc. Finally, the thermodynamic stability of these crystals is analyzed. The results show that Cs_2_SeI_6_, Rb_2_SeI_6_, Cs_2_SeBr_6_ and Rb_2_SeBr_6_ can be used as ideal candidates for light absorption materials. This work can not only guide experiments and design experiments rationally, but also reduce research and development costs, shorten research time, and improve the success rate of material design.

## Computational methods

In this work we discuss the crystal structure, electronic band structure, elastic modulus and optical properties of perovskite crystals in detail. The calculations in this paper are mainly performed by the plane wave pseudopotentials method as implemented in Cambridge serial total energy package (CASTEP) in Material Studio^33–36^, and the conduction band and valence band edge positions are calculated with the DMOL^3^ module^37,38^. The theoretical basis of both computational modules is density functional theory (DFT). Further, the generalized gradient approximation (GGA) within the Perdew–Burke–Ernzerhof (PBE) functional is used for the evaluation of exchange–correlation interactions for two computational modules. The lattice parameters of the crystal are shown in Fig. 4, and k pionts of supercell are set as 2 × 2 × 2. The calculation of the Dmol^3^ software package adopts the numerical basis set, which is set as 4.4 Double Numerical plus d-functions basis (DND). The accuracy and qualities are selected as "fine". SCF tolerance is 10^–5^ eV/atom for perovskite crystals. All the other sets are the default. While the calculation of CASTEP uses plane waves basis set. The electronic configurations 5s^1^, 5s^2^5p^2^, 3s^2^3p^5^, 4s^2^4p^5^ and 5s^2^ 5p^5^ are considered in valence for Rb, Sn, Cl, Br and I atoms, respectively. And the cut-off energy is set to 500 eV. The structure optimization process stops until the atomic Hellmann–Feynman force is less than 0.01 eV/Å. The energy convergence criterion is set to 10^–5^ eV.

## Model for perovskite solar cells

**Figure 1:**
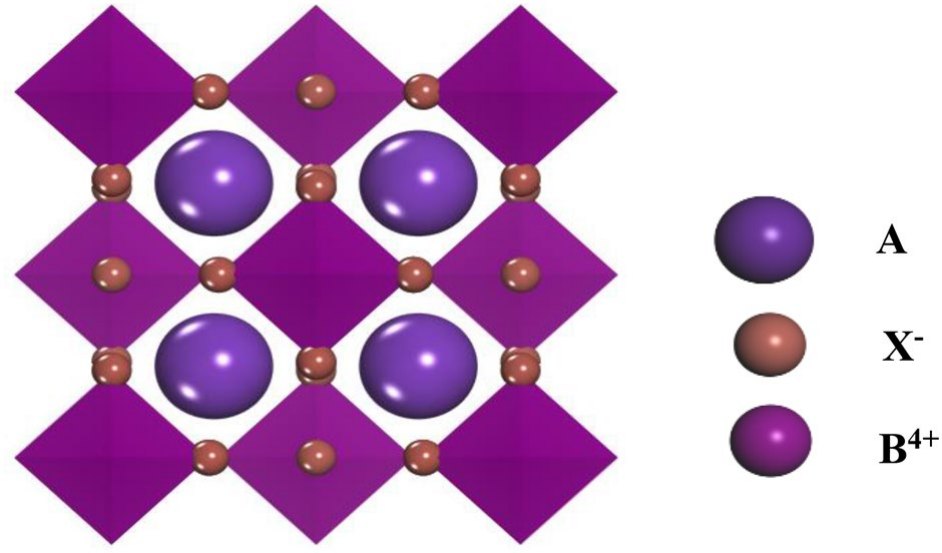
The crystal structure model of A_2_BX_6,_ which is double perovskites have high-symmetric cubic.

**Figure 2:**
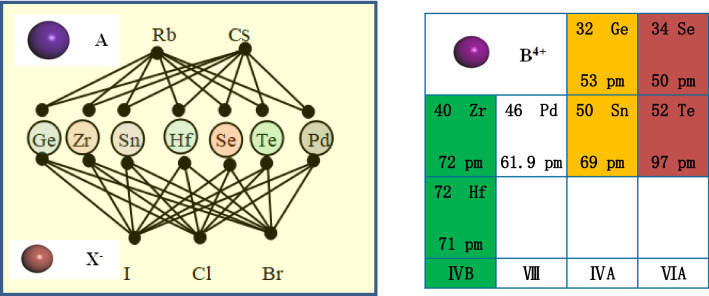
(**a**) High-throughput screening methods for network chain structure diagram and (**b**) properties of important elements germanium (Ge), selenium (Se), zirconium (Zr), palladium (Pd), tin (Sn), hafnium (Hf), tellurium (Te).

In this section, we consider inorganic halide double perovskite A_2_BX_6_ and its crystal structure (shown in Fig. 1), where A is a monovalent cation, B is a tetravalent cation, and X is a halide anion) type perovskite. For perovskite compounds, the octahedral factor is used to predict the formation of BX_6_ octahedra, and the tolerance factor is used to predict calcium formation and distortion of the titanite structure empirically. Likewise, in the perovskite crystal-derived structure A_2_BX_6_, we can combine the octahedral factor and the radius ratio to predict the formation and deformation of the structure, where A = Cs, Rb, B = Ge, Zr, Sn, Hf, Se, Te and Pd, and the halide anions X = I, Cl, Br. High-throughput screening methods and properties of important elements is shown in Fig. 2. The small octahedral factor indicates that it is not favorable for the formation of BX_6_ octahedra. Small radius ratios lead to evacuated cavities and lower structural symmetry, even different connectivity of fully octahedral networks. Each crystal structure parameters of these compounds are obtained from the International Crystal Structures of Database (ICSD) synthesized and characterized by experiments^39,40^.

To evaluate the structural stability of materials in perovskite structures, we further explore the empirical tolerance factor of them, which is an important parameter for evaluating whether cations in perovskite materials can be substituted. This factor has been widely used by previous researchers^41^ in the current double perovskite A_2_BX_6_, and the effective^42,43^ tolerance factor t could be defined as

1 $$ t = \frac{{R_{A} + R_{X} }}{{\sqrt 2 \left[ {R_{B} + R_{X} } \right]}}. $$

**Figure 3:**
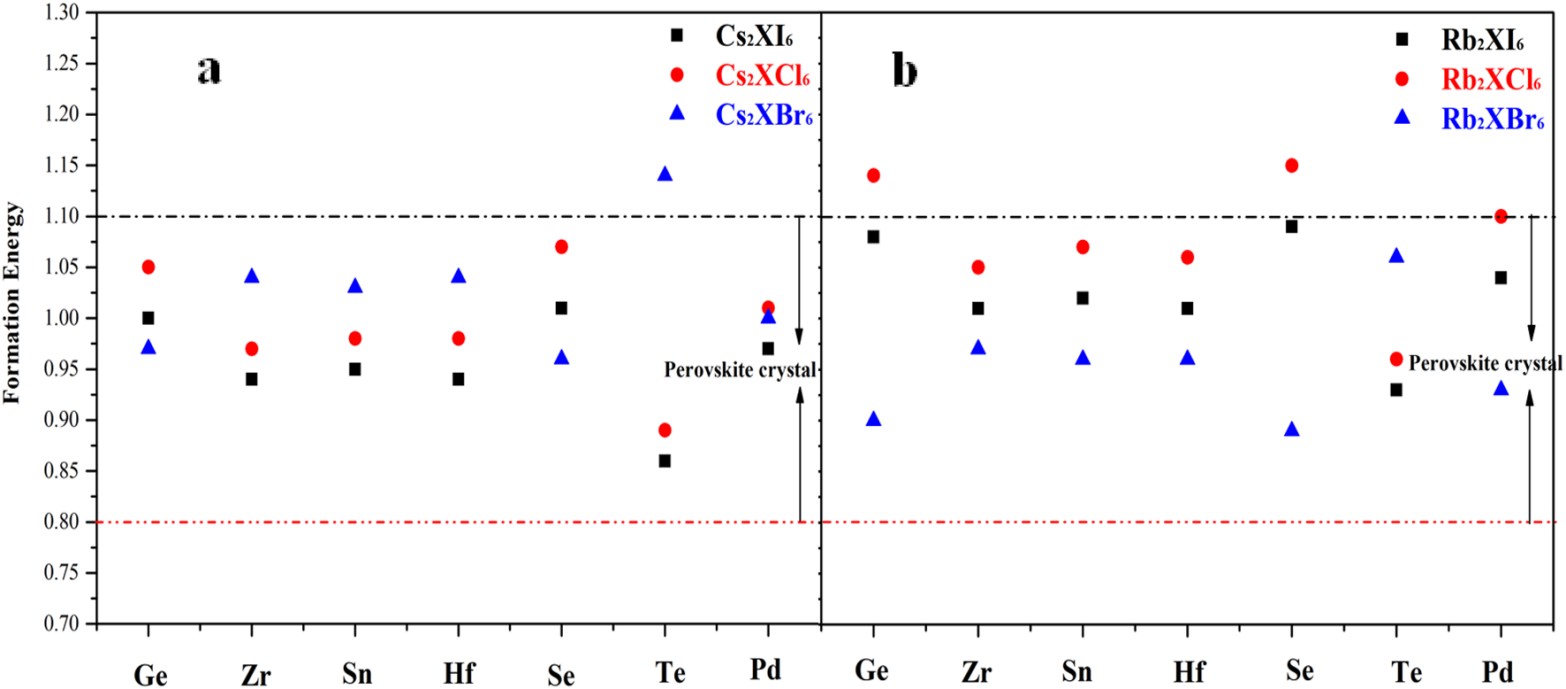
Tolerance factor of (a) Cs_2_BX_6_ and (b) Rb_2_BX_6_.

In the formula, if there are multiple ions in A and B position, the average radius can be taken. It is a semi-empirical formula that can roughly describe the stability of the perovskite structure. Studies have shown that the tolerance factor of structurally stable perovskite compounds are generally between 0.78 and 1.10. In general: (1) When tolerance factor t is close to 1.0, the compound has an equiaxed Pm3m structure. (2) If tolerance factor t deviates greatly from 1.0, other structures would be formed, in which R_Cs_ = 167 pm, R_Rb_ = 152 pm, R_I_ = 220 pm, R_Cl_ = 181 pm, R_Br_ = 196 pm. The calculation result of tolerance factor t is shown in Fig. 3.

**Figure 4:**
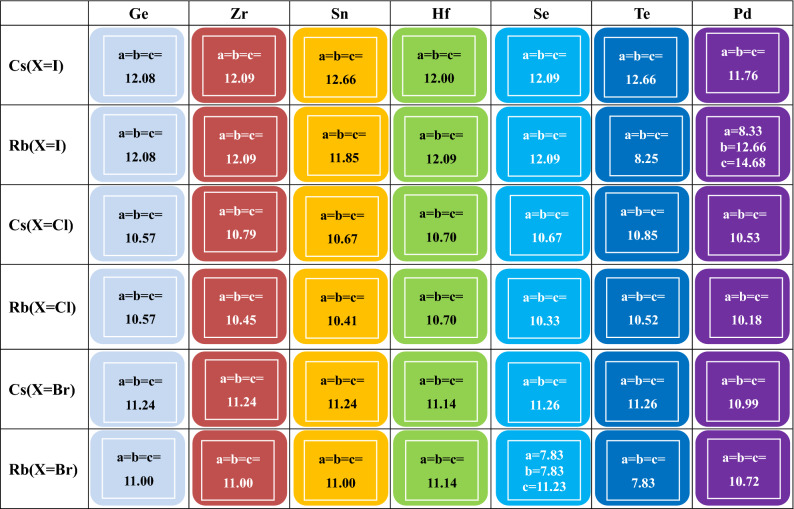
Lattice parameters of A_2_BX_6_.

The results show that only three tolerance factors of Rb_2_GeI_6_, Rb_2_SeI_6_, Cs_2_TeI_6_ are (1.14, 1.15, 1.14) slightly larger than 1.10, and the tolerance factors of other crystals are all between 0.8–1.10. The results show that these combinations are in line with the properties of perovskite compound materials. Next, the lattice parameters of these crystals are analyzed, and all possible combinations of crystal compounds are constructed based on the structure of the crystals by using MS software, and the crystal parameters are optimized which is shown in Fig. 4, with the unit (Å) for crystal bond length a, b, or c.

**Figure 5:**
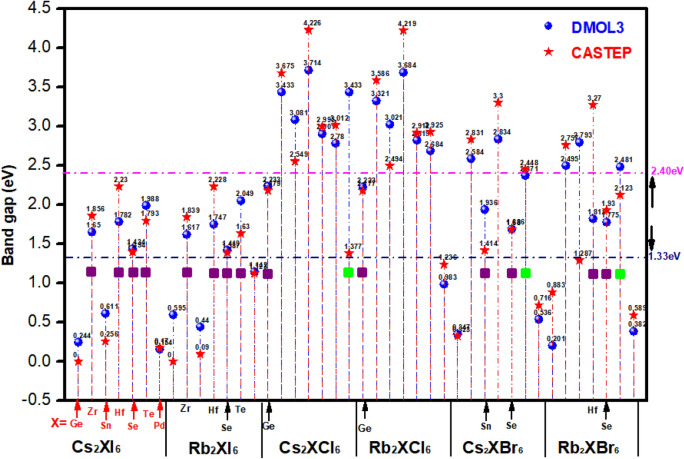
The value of bandgaps for A_2_BX_6_.

The value of the band gap of the crystal material is important factor affecting the light absorption efficiency of the perovskite layer. As we know that the smooth transition of electrons from the valence band to the conduction band can be achieved only when the photon energy $h\nu$ is greater than the band gap of the crystal. In order to analyze the optical properties of these materials, we calculated the band gaps of 42 kinds of materials with CASTEP and DMOL^3^ modules, as shown in Fig. 5 refer to the calculation results of some general perovskite materials in other papers ref. Table 1, our calculated band gap results are well agree with them.

**Table 1:** Some known band gap reference values of perovskite crystals.

Name	This result	Reference	Name	This result	Reference
Cs_2_SeCl_6_	2.99	2.95 eV^44^	Cs_2_HfBr_6_	3.30	3.3 eV^46^
Cs_2_TeCl_6_	3.07	3.10 eV^44^	Cs_2_HfI_6_	2.23	2.18 eV^46^
Cs_2_SeBr_6_	1.68	2.64 eV ^45^	Cs_2_HfCl_6_	4.226	4.12 eV^46^
Cs_2_SeI_6_	1.394	1.15 eV ^45^	Cs_2_ZrI_6_	1.856	1.92 eV^47^

It can be seen from Fig. 5 that the value of band gap for 14 kinds of crystals are between 1.33 eV and 2.40 eV, such as Cs_2_ZrI_6_, Cs_2_HfI_6_, Cs_2_SeI_6_, Cs_2_TeI_6_, Rb_2_ZrI_6_, Rb_2_HfI_6_, Rb_2_SeI6, Rb_2_TeI_6_, Cs_2_GeCl_6_, Rb_2_GeCl_6_, Cs_2_SnBr_6_,Cs_2_SeBr_6_, Rb_2_HfBr_6_, Rb_2_SeBr_6_, they are more suitable as light-absorbing layer materials. The band gap size of the iodine-containing halide perovskite material shows relatively excellent visible light absorption performance, and its performance is better than that of the halide perovskite material containing Br and Cl.

## Electronic properties of A_2_BX_6_

**Figure 6:**
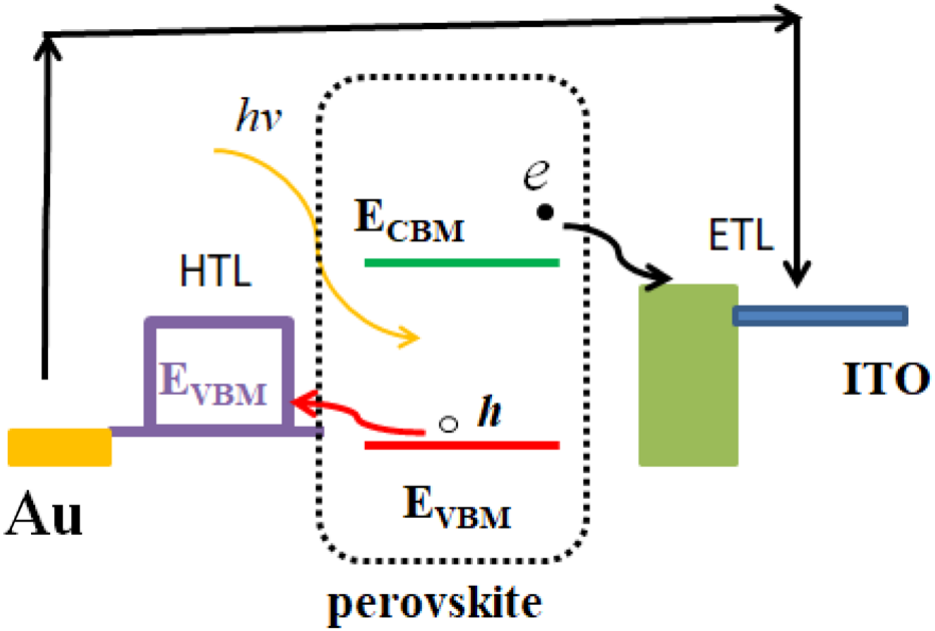
The device configuration and working principle of perovskite solar cells.

As a photoelectric conversion material for solar cells, the electronic structure of A_2_BX_6_ is a key factor affecting spectral absorption. Therefore, we further analyze the energy band structures of these materials, and we compared the electric potential of the back electrode of the hole transport layer in the conducting glass in perovskite solar cells. Figure 6 shows that the conduction band position of the perovskite is higher than the conduction band potential value of the electron transport layer, and the valence band position is lower than the potential value of the hole transport layer, which is more conducive to the separation of electrons and holes and forms a stable potential difference external power supply.

The perovskite light-absorbing layer absorbs solar photons, and electrons in the valence band obtained energy transitions to the conduction band. And holes are left in the valence band, so the electrons and holes are separated. The maximum value of the valence band of the perovskite material is lower than that of the holes transport material, while the minimum value of its conduction band is higher than that of the electron transport material. In this way, smooth separation of carriers can be easily achieved.

**Figure 7:**
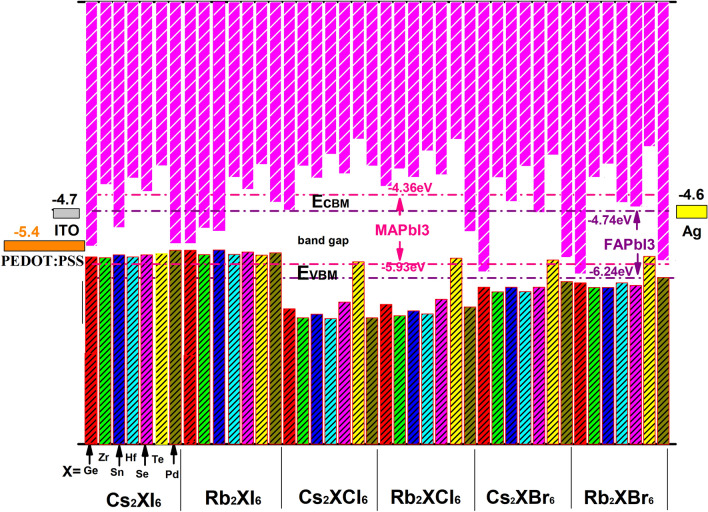
The valence band maxima (VBM) and conduction band minima (CBM) of 42 crystals, the energy band value of ITO, Ag and PEDOT:PSS is shown in Figure with unit (eV) .

We calculate the valence band maxima (VBM) and conduction band minima (CBM) of 42 compounds and compared them with the band gaps of two efficient organic/inorganic perovskite materials (CH_3_NH_3_PbI_3_) MAPbI_3_, (NH_2_CH = NH_2_PbI_3_) FAPbI_3_ form. It can be seen from Fig. 7 that the maximum value of the valence band of these materials and the size of the valence band of the electron transport layer material, the hole transport layer and the back electrode material are helpful to analyze the efficiency of carrier migration of these materials. We preferentially analyze the above 14 materials with band gaps between 1.33 and 2.40 eV. Considering the energy band value of ITO, Ag and PEDOT:PSS, it requires the minimum edge of the conduction band is greater than − 4.6 eV, and the maximum value of the valence band is lower than − 5.4 eV. There are 11 kinds of crystals that satisfy the above conditions as follows: Cs_2_ZrI_6_, Cs_2_HfI_6_, Cs_2_SeI_6_, Cs_2_TeI_6_, Rb_2_HfI_6_, Rb_2_SeI_6_, Rb_2_TeI_6_, Rb_2_GeCl_6_ Cs_2_SeBr_6_ , Rb_2_SeBr_6_ and Rb_2_HfBr_6_ . And we found that the conduction band and valence band edge value of the four crystals such as Cs_2_SeI_6_, Rb_2_SeI_6_, Cs_2_SeBr_6_, Rb_2_SeBr_6_ are relatively close to the values of (CH_3_NH_3_PbI_3_) MAPbI_3_ and (NH_2_CH=NH_2_PbI_3_) FAPbI_3._

### Optical properties of A_2_BX_6_

**Figure 8:**
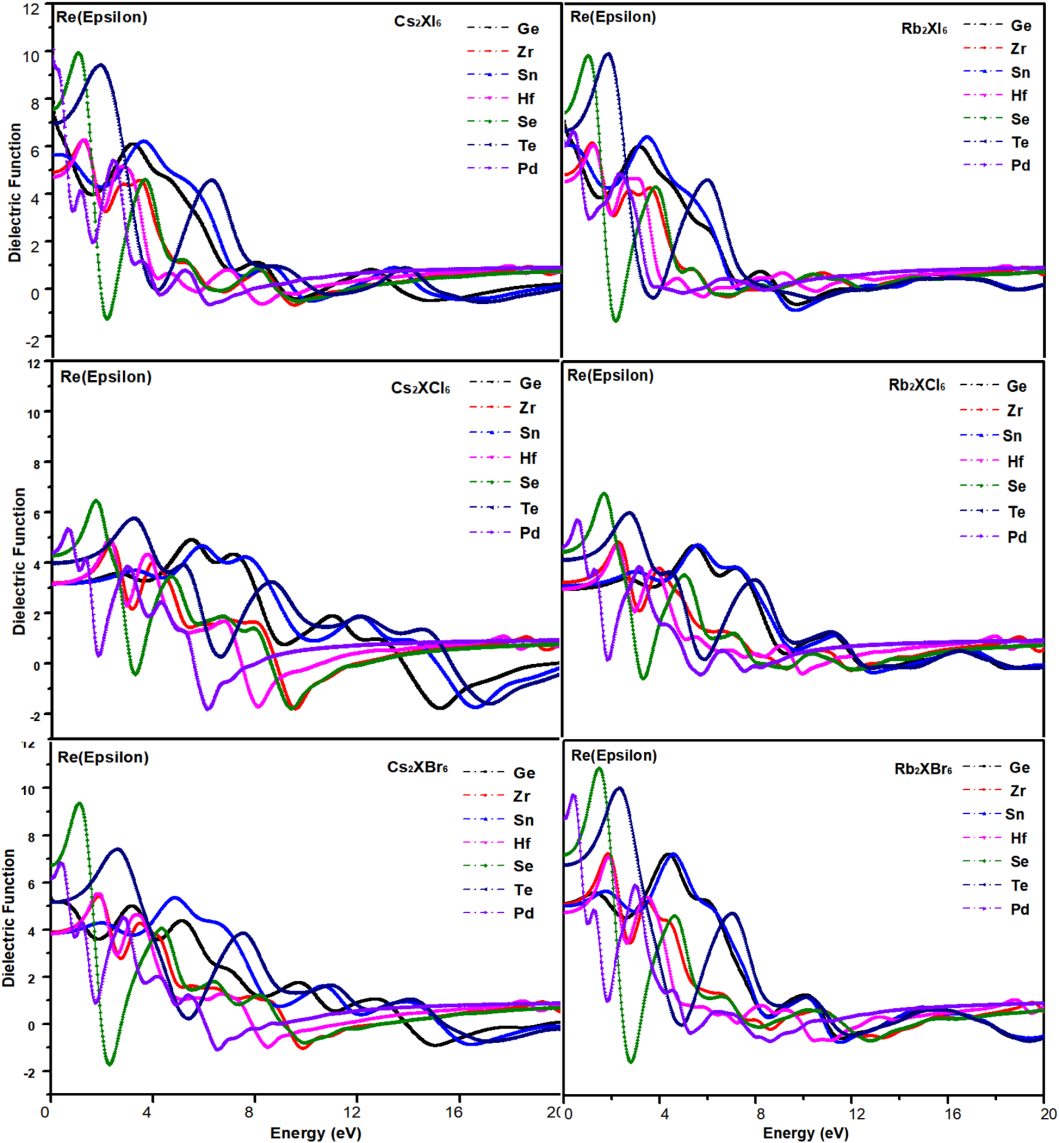
Real part characteristic curve of dielectric.

**Figure 9:**
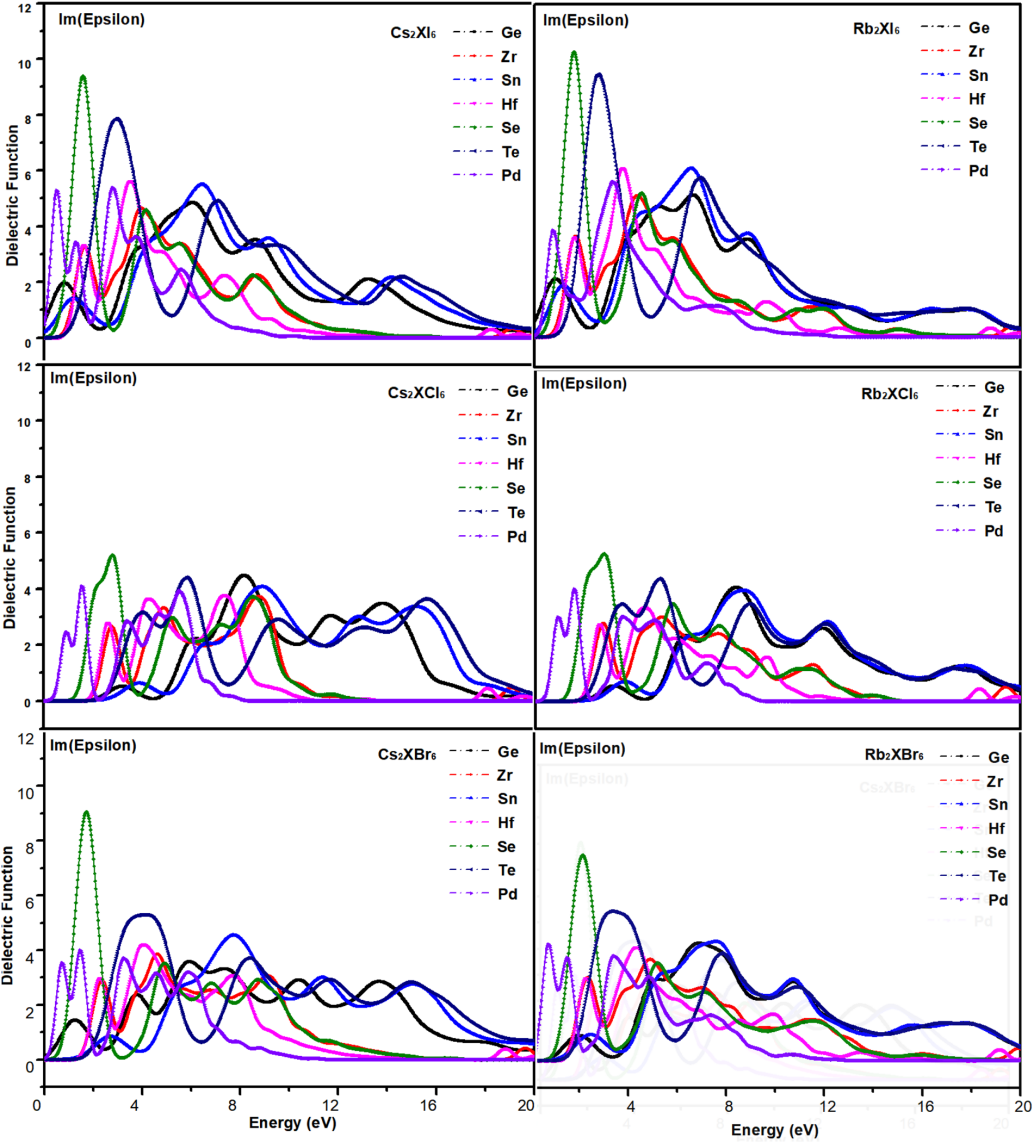
Imaginary part characteristic curve of dielectric.

The dielectric function ε(ω) is used to describe the linear response properties of photovoltaic materials to electromagnetic radiation, and is one of the effective indicators to reflect the spectral properties of photovoltaic materials. Its optical response properties can be described by the following complex dielectric function ε(ω)^48^

2 $$ \varepsilon (\omega ) = \varepsilon_{1} (\omega ) + i\varepsilon_{2} (\omega ) $$

where $\varepsilon_{1} (\omega ) = n^{2} - k^{2} ,$ $\varepsilon_{2} (\omega ) = 2nk$ (n and k are the reflection coefficient and extinction coefficient, respectively)ε_1_ (ω) and ε_2_ (ω) are the real and imaginary parts of the dielectric function, respectively, depending on the optical frequency. The characteristic curve of the real part of the dielectric function obtained by calculation is shown in Fig. 8, and the characteristic curve of the imaginary part is shown in Fig. 9. ω represents the frequency of light. The absorption coefficient is directly related to the band gap of the material. Optical properties can be obtained from complex dielectric functions. The absorption coefficient I (ω) can be derived from ε_1_(ω) and ε_2_(ω). The specific formula of the equation is shown as following^48^:

3 $$ I(\omega ) = \sqrt 2 \omega \left[ {\sqrt {\varepsilon_{1} (\omega ) + \varepsilon_{2} (\omega )} - \varepsilon_{1} (\omega )} \right]^{{{1 \mathord{\left/ {\vphantom {1 2}} \right. \kern-\nulldelimiterspace} 2}}} $$

**Figure 10:**
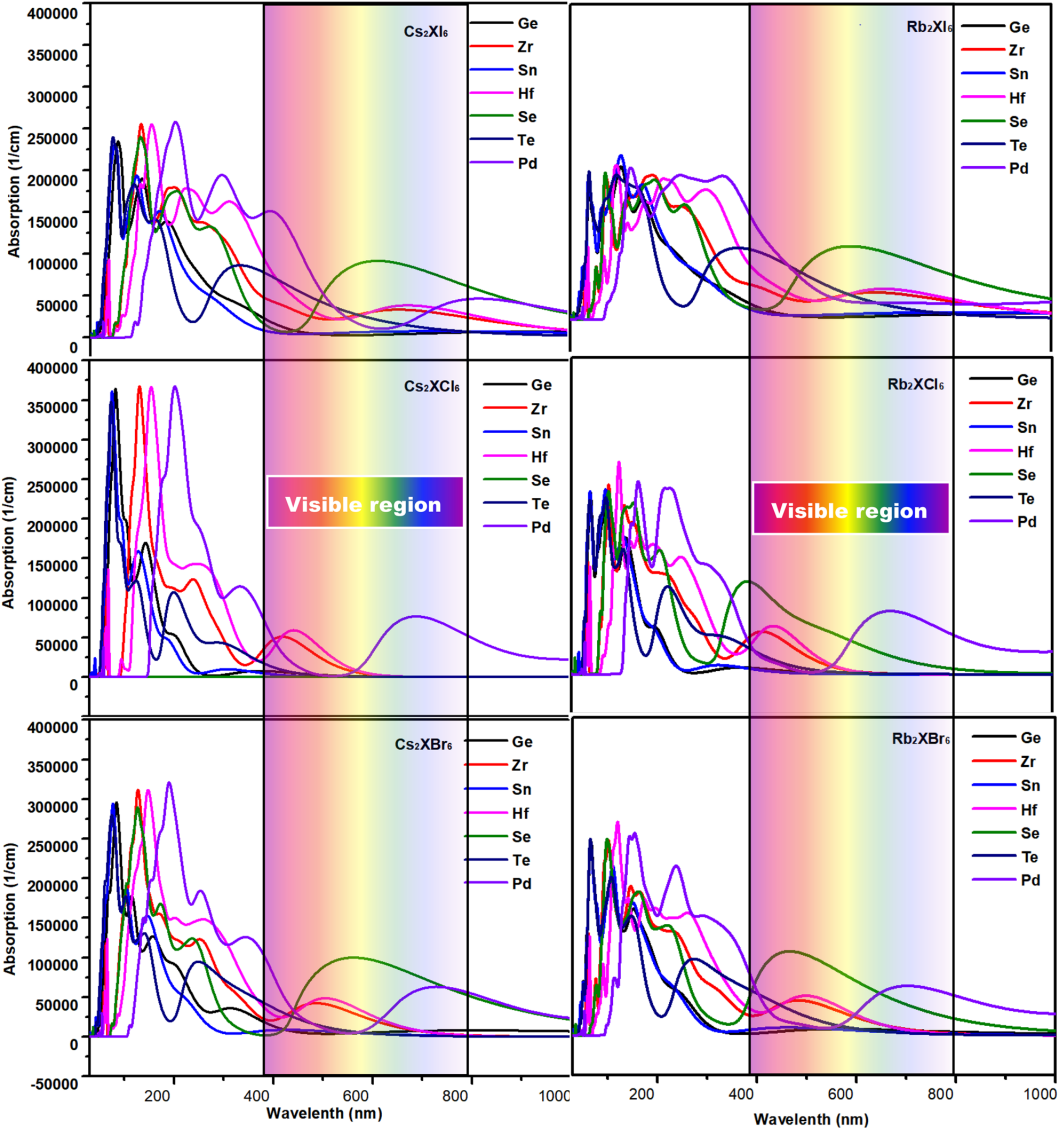
Absorption spectra of 42 crystalline materials.

Next, the optical absorption coefficients of these 42 compounds are calculated by the PBE method and are shown in Fig. 10. Absorption is seen at energies less than the bandgap, which is due to an error in the calculation method and did not affect our analysis of the results. The absorption coefficient is as high as 10^5^ cm^−1^, which is mainly due to the absorption between the p orbitals of halide ions Cl, Br, and I in the valence band and the s-orbital of Sn in the conduction band. The optical absorption coefficients of the three structures are shown in Fig. 10. When X changed from I^-^ to Cl^-^, the absorption spectrum of Cs_2_TeX_6_ is blue-shifted due to the band gap increase. Comparatively, A_2_XI_6_ has better absorption than A_2_XCl_6_ and A_2_XBr_6_. The light absorption curves of seven tetravalent ions (Ge, Zr, Sn, Hf, Se, Te, Pd) compounds can be seen, their light absorption peaks are concentrated in the ultraviolet region, and those with better absorption effect in the visible light region have Se and Pd-containing materials.

In contrast, the light absorption behavior of Rb_2_XCl_6_ in the visible region is poor, but the light absorption in the ultraviolet region is also considerable. Others with better light absorption are halide perovskite materials containing Hf and Zr. From the absorption spectrum, it can be seen that Cs_2_SeI_6_, Rb_2_SeI_6_, Cs_2_SeBr_6_, Rb_2_SeBr_6_, Rb_2_PdBr_6_, Cs_2_PdBr_6_ are potential candidates for the absorption layer of solar cells. Comparing with the Figs. 5 and 7, we can find that the conclusion obtained according to the energy band theory is in good agreement with the absorption spectrum Fig. 10.

### Electronic properties of A_2_BX_6_

**Figure 11:**
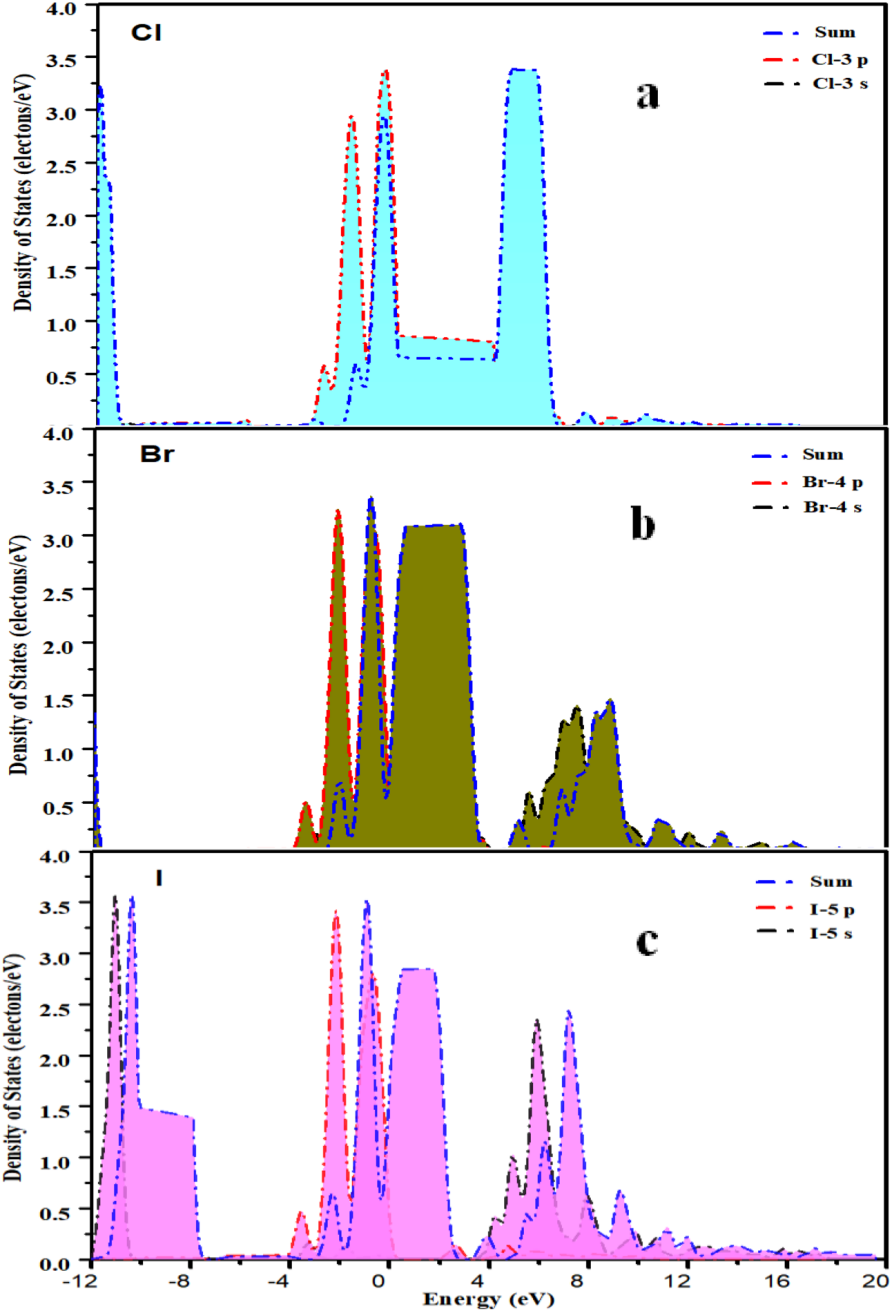
(**a**–**c**) show the PDOS curve of Halogen element chlorine (Cl), bromine (Br), iodine (I) respectively.

Among halogen elements, as the atomic number increases, the ability of atoms to attract the outermost electrons gradually decreases, so the ability to obtain electrons decreases with the increase of atomic number. It can be seen from the PDOS curve in Fig. 11a–c that the curve peaks of Cl, Br, and I proceed in the direction of decreasing energy, and the energy of some electrons concentrate in the energy band decreases continuously.

**Figure. 12:**
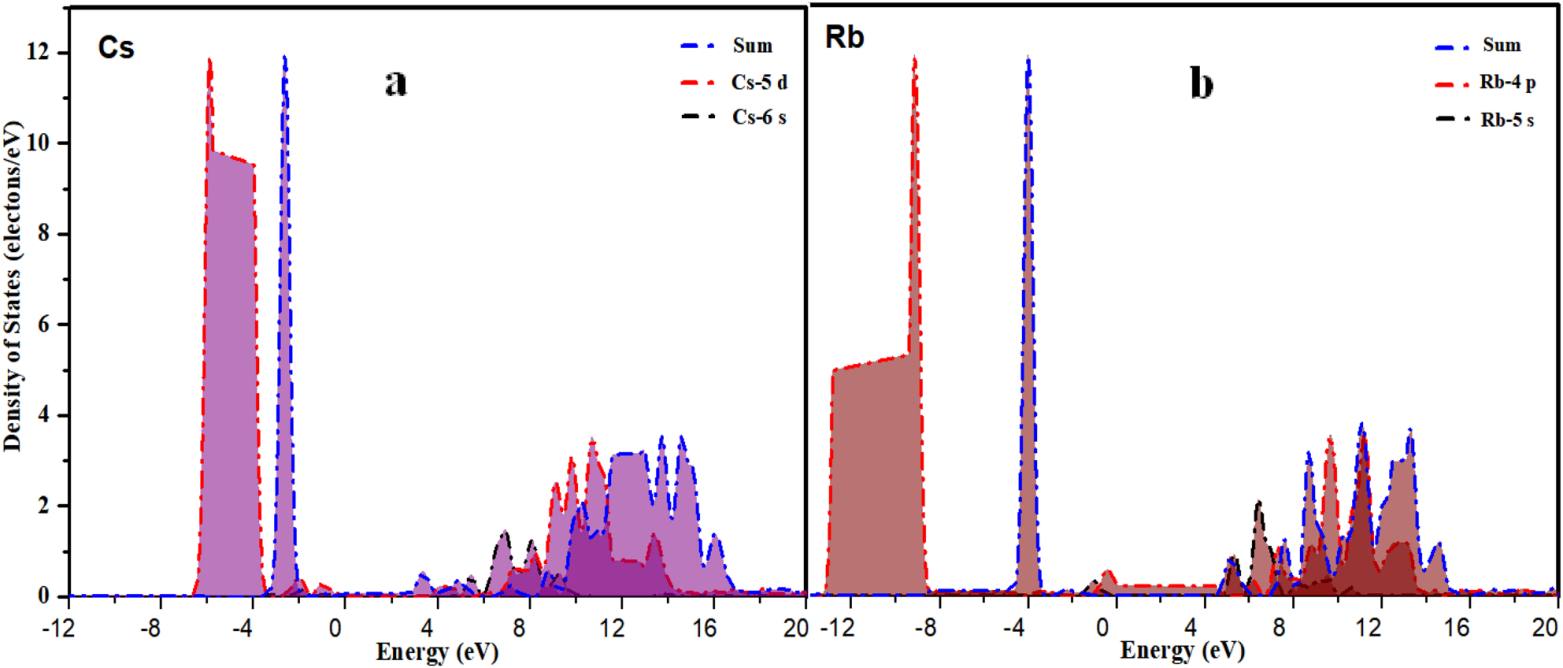
PDOS curves of (**a**) Cs and (**b**) Pd.

Figure 12a,b illustrate the partial density of states (PDOS) for Cesium (Cs) and Rubidium (Rb). And the contribution to Rb mainly came from 5 s-orbital and 4p-orbital electrons, the element Cs mainly comes from 6 s-orbital electrons and 5d-orbital electrons. It can be seen from the Fig. 12a,b that they have no band gap, which shows that they have obvious metallicity. From the position in the periodic table of elements, Rb and Cs are at the same group (IA). The difference of them is that Rb located in the 5th periods and Cs in 6th periods. There are 55 electrons in outer cores for Cs, and 37 electrons in outer Rb cores. But their outermost layer has only one electron, which is easy to lose and become a positive monovalent ion. In contrast, the compositions of PDOS curves for the two elements are very similar. Nevertheless the electronic density of states of rubidium is more concentrated. Further analysis shows that the 6 s-orbital electrons of Cs and the 5 s-orbital electrons of Rb did not contribute to their low energy bands. Above appearance may help us to comprehend the fact of difference in the effective mass between hole and electron.

**Figure 13:**
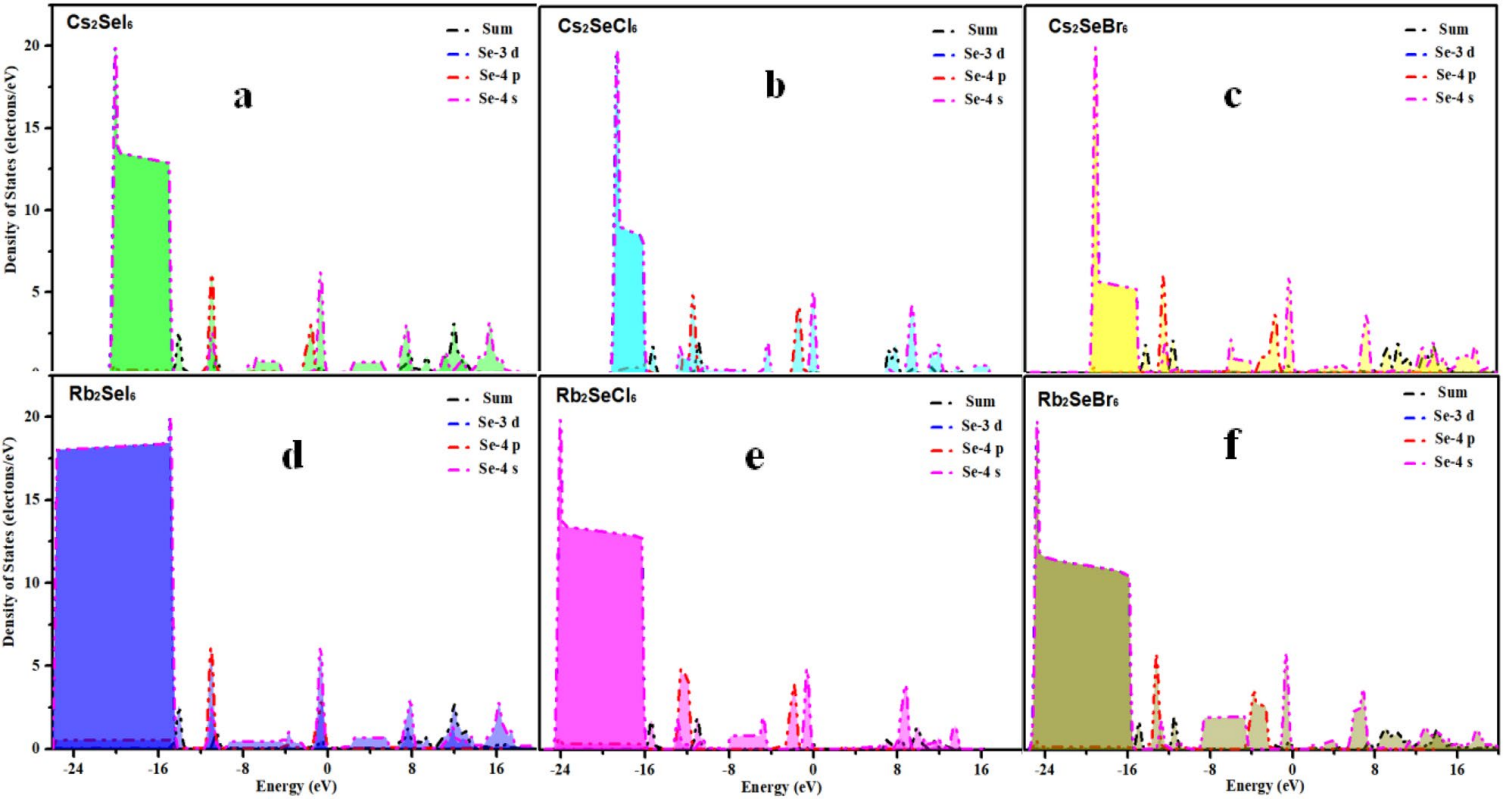
PDOS curves of Se-containing perovskite materials (**a**) Cs_2_SeI_6_ (**b**) Cs_2_SeCl_6_ (**c**) Cs_2_SeBr_6_ (**d**) Rb_2_SeI_6_ (**e**) Rb_2_SeCl_6_ (**f**) Cs_2_SeBr_6_.

Since selenium(Cs)-containing perovskite compounds have good light absorption properties and band gap structures, this paper uses selenium-containing compounds as an example to calculate the PDOS curves of six crystals, involving electrons 3d^10^ 4s^2^ 4p^6^. It can help us to analyze the ability of Cs to lose electrons when it bonds with different halogens. The PDOS curves of Fig. 13a–f suggest that with the increase of atomic radii of Cl, Br and I, the number of exonuclear electrons increases correspondingly, and the binding ability of the nucleus and electrons decreases. Further, we found that the ability to obtain electrons decreases and their oxidizing property decreases. Longitudinal comparison we found that when Se combined with Rb to form a crystal, the area covered by the density of states would be widen.

## Stability of perovskite crystals A_2_BX_6_

### Thermodynamical stability of A_2_BX_6_

To explore the thermodynamic stability of these crystals, we calculate their formation energies. For perovskite materials, the formation energy can be calculated using the following expression^49^:

4 $$ \Delta E_{1} = E(A_{2} BX_{6} ) - 2E(AX) - E(BX_{4} ) $$

**Figure 14:**
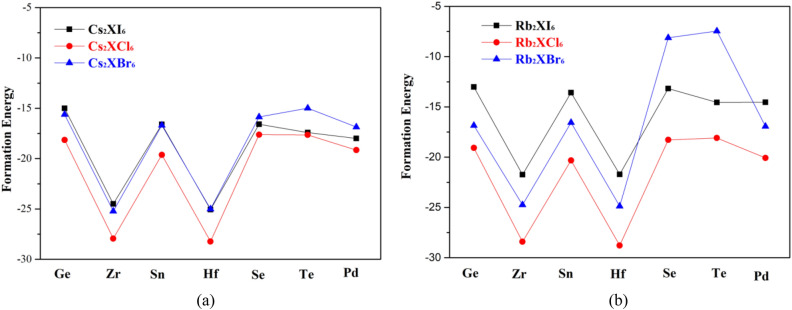
The curve trend Figure of formation energy (**a**) Cs_2_X (I, Cl, Br)_6_, (**b**) Rb_2_X (I, Cl, Br)_6_.

In general, the initial state of compound formation is the elemental state of A and B, and the bond between A and B atoms need to be broken first, which requires absorbing a certain amount of energy. If we want to form compound AB again, energy need to be released. The energy releases and absorbs by the difference between the two is the heat of generation, which is equivalent to the reaction heat of a chemical reaction. The more negative the formation energy, the more stable the compound. The calculation results are shown in Fig. 14a,b. The formation of compounds containing Cs and Rb elements is roughly the same, and the perovskite crystals containing Cs are more stable. Among these elements with positive tetravalent ion, there are also Zr and Hf perovskite. The crystal structure is more stable. If the band gap size and light absorption properties of Cs_2_SeI_6_, Rb_2_SeI_6_, Cs_2_SeBr_6_ and Rb_2_SeBr_6_ are considered comprehensively, they can be considered as ideal candidates for light absorption materials. In addition, Cs_2_ZrI_6_, Cs_2_HfI_6_, Rb_2_ZrI_6_, Rb_2_HfI_6_, Rb_2_HfBr_6_ are also superior in stability and band gap structure. Their light absorption behavior in the visible region is poor, but their light absorption in the ultraviolet region is also considerable.

### Mechanical properties of A_2_BX_6_

Then, the mechanical stability of these materials is predicted from the calculated elastic moduli, which is calculated using finite strain theory^50^. The cubic crystal has three independent elastic constants (C_11_, C_12_ and C_44_).The natural mechanical stability criterion is given by: C_11_–C_12_ > 0, C_11_ + 2C_12_ > 0, C_44_ > 0. Then we calculate elastic constants of the 42 inorganic double perovskites are shown in Tables 1, 2 and 3. According to the above criteria, these compounds are mechanically stable. To differentiate between ductile and brittle materials, Pugh's ratio (B/G) and Poisson's ratio (v) are used. Poisson's ratio can be evaluated using the following formula:

5 $$ \nu = (3B - 2G)/(6B + 2G) $$

where B and G are the bulk modulus and shear modulus, respectively. The critical B/G and values are 1.75 and 0.26 respectively^38,44^. In other words, the composite is considered to be a ductile material when the B/G value is greater than 1.75 or the υ value is greater than 0.26, otherwise it is considered a brittle material. The calculation results are shown in Tables 2, 3 and 4. The calculated value of elastic constants C_ij_, bulk modulus B (GPa), shear modulus G (GPa), Young’s modulus E (GPa), Pugh’s ratio K/G, and Poisson ratio (ν) of cubic perovskite, Averaged sound velocity V(m/s).

**Table 2:** Elastic Modulus of Iodide Inorganic Perovskites.

	C_11_ (GPa)	C_12_ (GPa)	C_44_ (GPa)	B (GPa)	E (GPa)	G (GPa)	ν	V (m/s)
Cs_2_GeI_6_	10.60 ± 0.11	1.45 ± 0.09	6.17 ± 0.001	4.48 ± 0.037	11.77	5.54	0.062	1250.65
Cs_2_HfI_6_	14.86 ± 0.39	3.17 ± 0.89	7.71 ± 0.005	7.06 ± 0.342	15.73	6.96	0.128	1336.07
Cs_2_PdI_6_	11.73 ± 1.15	4.21 ± 1.63	6.19 ± 0.21	6.00 ± 0.56	11.7	4.94	0.19	1109.36
Cs_2_SnI_6_	11.64 ± 1.67	2.08 ± 0.24	8.32 ± 0.001	5.27 ± 0.31	14.41	6.90	0.043	1338.68
Cs_2_TeI_6_	12.93 ± 1.54	2.81 ± 1.29	6.85 ± 0.002	6.18 ± 0.55	13.83	6.18	0.13	1289.24
Cs_2_ZrI_6_	12.86 ± 0.21	2.27 ± 0.76	6.90 ± 0.016	5.84 ± 0.29	13.92	6.30	0.10	1330.93
Cs_2_SeI_6_	11.49 ± 0.27	0.64 ± 0.19	5.97 ± 0.001	4.29 ± 0.10	11.91	5.74	0.037	1275.55
Rb_2_GeI_6_	9.25 ± 0.188	0.88 ± 0.10	5.32 ± 0.003	3.62 ± 0.05	10.01	3.63	0.04	1219.06
Rb_2_HfI_6_	11.07 ± 0.44	0.59 ± 0.89	6.35 ± 0.006	4.028 ± 0.399	11.72	5.76	0.018	1265.34
Rb_2_PdI_6_	8.32 ± 0.435	4.25 ± 0.88	2.56 ± 0.129	6.33 ± 0.421	10.69	4.25	0.26	979.59
Rb_2_SeI_6_	10.38 ± 0.185	0.75 ± 0.177	4.99 ± 0.001	3.94 ± 0.080	10.43	4.92	0.059	1238.07
Rb_2_SnI_6_	12.72 ± 0.634	3.03 ± 1.10	8.64 ± 0.003	6.21 ± 0.435	15.39	7.07	0.088	1377.09
Rb_2_TeI_6_	11.23 ± 4.063	1.69 ± 2.92	1.15 ± 1.23	7.92 ± 2.19	3.36	1.19	0.407	0
Rb_2_ZrI_6_	9.95 ± 1.86	1.08 ± 0.184	6.02 ± 0.004	4.316 ± 0.322	11.77	5.59	0.05	1303.08

**Table 3:** Elastic Modulus of Chloride Inorganic Perovskites.

	C_11_ (GPa)	C_12_ (GPa)	C_44_ (GPa)	B (GPa)	E (GPa)	G (GPa)	ν	V (m/s)
Cs_2_GeCl_6_	16.63 ± 0.908	3.77 ± 2.916	6.48 ± 1.02	8.16 ± 1.052	15.34	6.45	0.188	1589.73
Cs_2_HfCl_6_	15.71 ± 3.088	6.09 ± 2.444	10.99 ± 0.025	7.95 ± 0.968	19.48	8.91	0.094	1672.25
Cs_2_PdCl_6_	14.88 ± 0.852	0.91 ± 0.979	6.34 ± 0.824	5.69 ± 0.462	14.24	6.46	0.102	1511.75
Cs_2_SnCl_6_	15.21 ± 1.820	6.73 ± 0.655	10.99 ± 0.001	9.18 ± 0.452	19.71	8.63	0.142	1697.10
Cs_2_TeCl_6_	17.09 ± 1.723	3.28 ± 0.601	8.20 ± 0.002	8.11 ± 0.284	18.07	8.00	0.022	1744.96
Cs2ZrCl_6_	13.6 ± 0.501	4.47 ± 0.927	10.04 ± 0.002	7.33 ± 0.333	17.18	7.74	0.936	1659.38
Cs_2_SeCl_6_	13.30 ± 0.694	5.46 ± 0.274	5.98 ± 0.180	8.57 ± 0.516	13.11	5.22	0.255	1439.12
Rb_2_GeCl_6_	10.77 ± 0.144	3.93 ± 0.060	5.17 ± 0.112	5.98 ± 0.292	10.88	4.53	0.273	1442.78
Rb_2_HfCl_6_	13.48 ± 1.281	1.82 ± 0.959	9.16 ± 0.033	4.34 ± 0.794	14.07	6.79	0.584	1581.54
Rb_2_PdCl_6_	17.9 ± 2.626	6.31 ± 0.761	9.05 ± 0.833	9.95 ± 0.592	19.28	8.19	0.174	1774.54
Rb_2_SeCl_6_	14.86 ± 2.331	5.08 ± 0.340	6.70 ± 0.914	8.86 ± 0.581	15.56	6.42	0.212	1658.49
Rb_2_SnCl_6_	17.59 ± 2.979	7.71 ± 1.702	10.83 ± 0.057	11.59 ± 0.755	20.42	8.46	0.207	1774.97
Rb_2_TeCl_6_	16.78 ± 1.467	6.93 ± 1.054	8.53 ± 0.258	10.13 ± 0.523	18.76	7.84	0.196	1789.21
Rb_2_ZrCl_6_	18.72 ± 1.255	6.51 ± 2.577	11.05 ± 0.001	9.13 ± 1.043	9.129	7.97	0.172	1747.20

**Table 4:** Elastic moduli of bromide inorganic perovskites.

	C_11_ (GPa)	C_12_ (GPa)	C_44_ (GPa)	B (GPa)	E (GPa)	G (GPa)	ν	V (m/s)
Cs_2_GeBr_6_	11.17 ± 2.401	4.26 ± 0.518	8.923 ± 0.006	6.41 ± 0.383	14.89	6.68	0.114	1342.40
Cs_2_HfBr_6_	15.89 ± 0.435	4.63 ± 1.470	11.63 ± 0.003	7.41 ± 0.569	19.74	9.21	0.072	1496.64
Cs_2_PdBr_6_	22.70 ± 6.342	− 0.16 ± 1.109	5.61 ± 0.543	7.48 ± 1.074	18.02	8.19	0.519	1463.65
Cs_2_SnBr_6_	17.36 ± 0.393	3.01 ± 0.495	8.90 ± 0.006	8.01 ± 0.186	18.40	8.23	0.118	1555.68
Cs_2_TeBr_6_	13.02 ± 0.186	2.39 ± 0.128	8.53 ± 0.001	6.09 ± 0.067	15.75	7.36	0.069	1446.08
Cs_2_ZrBr_6_	15.18 ± 0.744	2.54 ± 1.790	9.09 ± 0.029	6.85 ± 0.706	17.08	7.78	0.098	1505.36
Cs_2_SeBr_6_	14.28 ± 0.741	2.03 ± 1.049	7.81 ± 0.001	5.98 ± 0.492	14.75	6.76	0.278	1424.76
Rb_2_GeBr_6_	10.81 ± 1.915	4.08 ± 0.823	9.29 ± 0.002	6.324 ± 0.433	15.23	6.93	0.099	1402.02
Rb_2_HfBr_6_	15.11 ± 3.474	2.61 ± 1.006	8.49 ± 0.018	6.52 ± 0.718	16.25	7.48	0.158	1481.47
Rb_2_PdBr_6_	13.54 ± 0.748	6.75 ± 0.923	8.13 ± 0.687	8.50 ± 0.492	15.61	6.49	0.201	1325.17
Rb_2_SeBr_6_	9.79 ± 0.535	2.27 ± 0.878	7.28 ± 0.002	5.06 ± 0.408	11.23	4.90	0.146	1195.87
Rb_2_SnBr_6_	18.77 ± 0.152	5.23 ± 0.673	9.19 ± 0.010	9.79 ± 0.266	19.29	8.23	0.111	1600.55
Rb_2_TeBr_6_	11.95 ± 2.248	4.02 ± 0.322	2.83 ± 0.536	7.58 ± 0.359	8.44	3.19	0.363	1014.96
Rb_2_ZrBr_6_	13.68 ± 2.566	5.48 ± 0.863	9.27 ± 0.003	8.69 ± 0.599	17.85	7.69	0.464	1541.25

**Figure 15:**
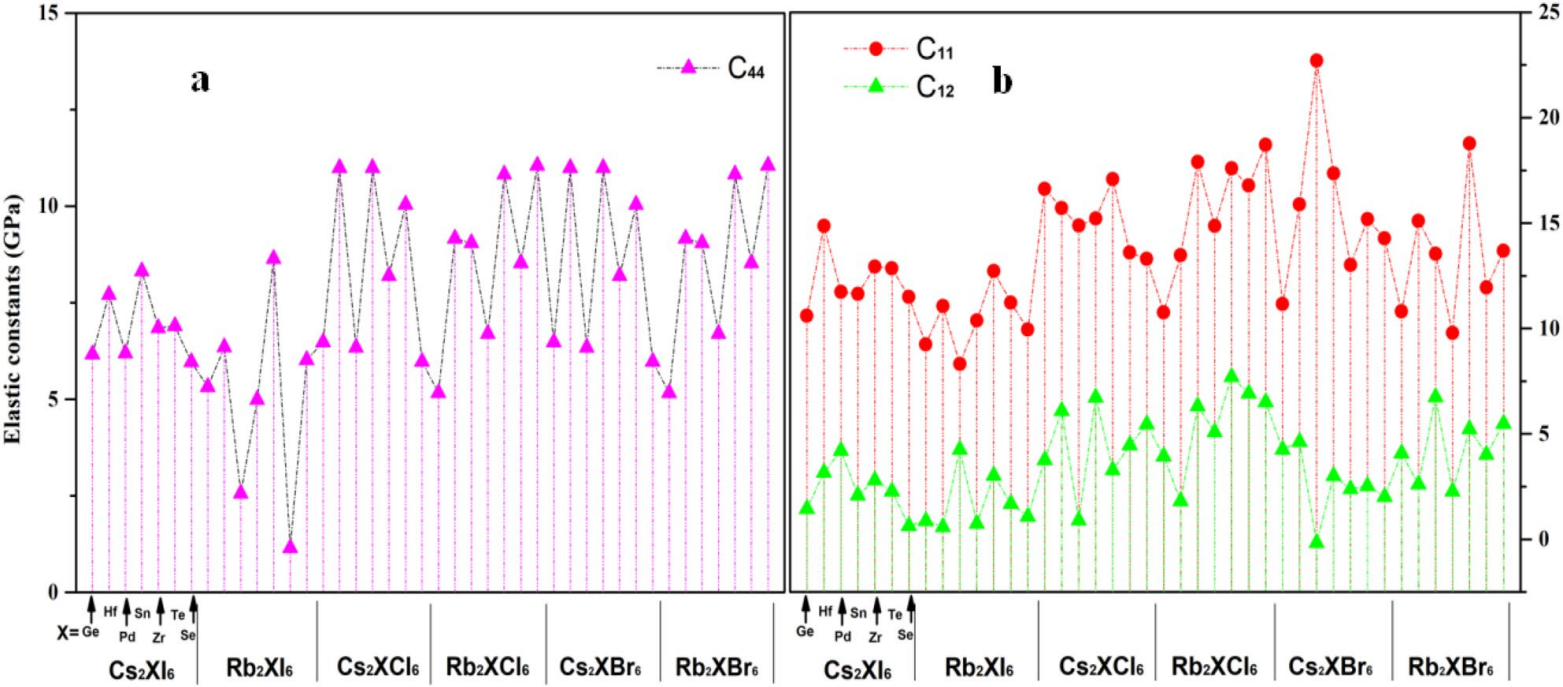
The intuitive graphics of the three components of elastic modulus are (**a**) C_44,_ (**b**) C_11_, C_12_ respectively, and they can show the elastic modulus along different axes of the crystal.

In order to analyze the stability of the crystal in more detail, we plot three elastic constants C_11_, C_12_ and C_44_ sufficient to explain the complete mechanical behavior of cubic symmetry as shown in Fig. 15. It is easy to conclude that they meet the criteria of C_11_-C_12_ > 0, C_44_ > 0, and C_11_ + 2C_12_ > 0. It indicates that their mechanical stability is relatively good.

**Figure 16:**
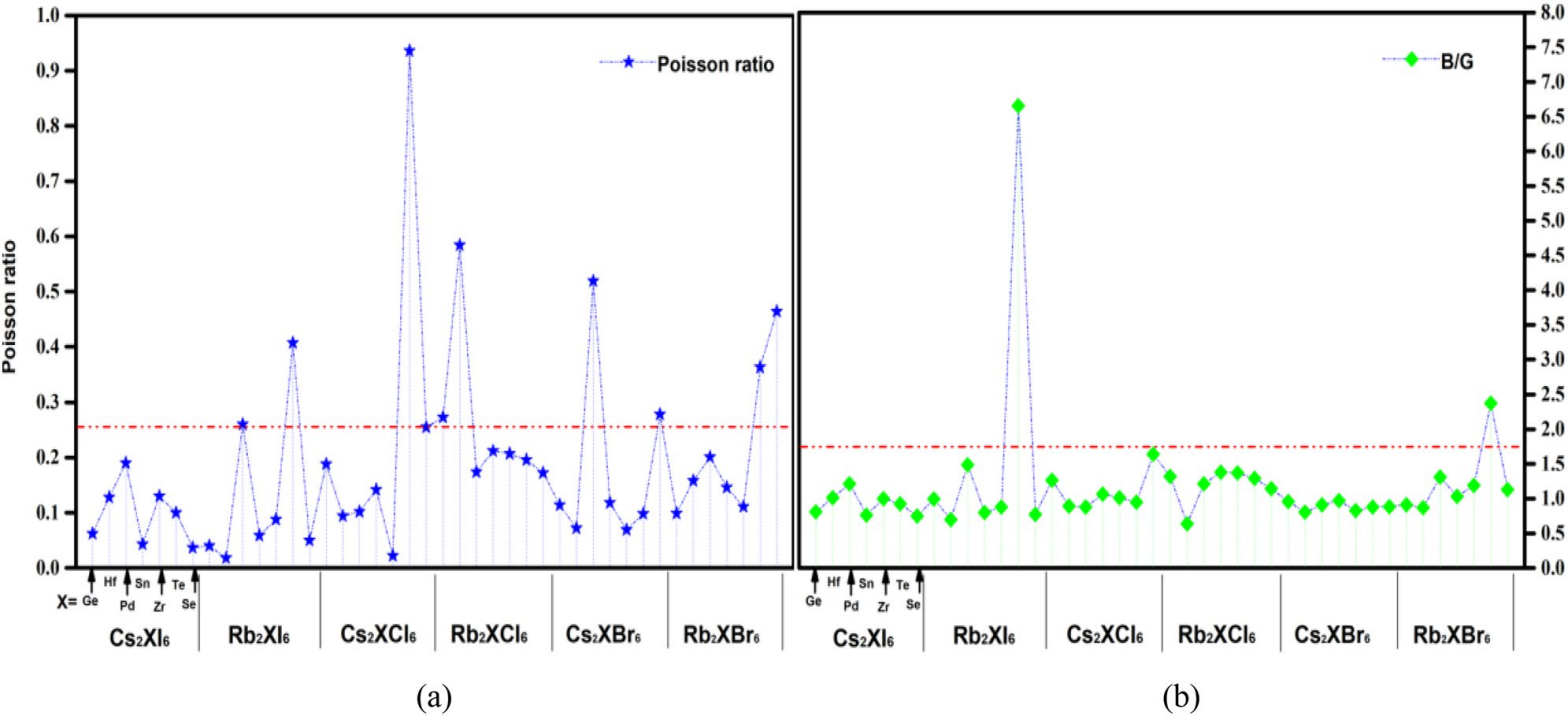
(**a**) Intuitive plot of Poisson's ratio for 42 kinds of inorganic double perovskite materials, the figure shows that the Poisson ratio of Cs_2_TeCl_6_ is the largest, (**b**) shows the B/G of Rb_2_TeI_6_ is the 42 kinds of them.

The critical limit of 1.75 for the Pugh ratio (B/G) distinguishes the brittle (B/G < 1.75) and ductile (B/G > 1.75) behavior of the studied material. The values report in Tables 1, 2 and 3 show the ductility behavior, the Poisson's ratio (υ) further ensures ductility, and the critical limit for ductile materials is υ > 0.26^50,51^. Figure 16 is a graph showing its Poisson's ratio and Pugh ratio, and the comparison shows that Rb_2_TeI_6_, Cs_2_TeI_6_, Rb_2_HfCl_6_, Cs_2_PdBr_6_, Cs_2_SeBr_6_, Rb_2_TeBr_6_, Rb_2_SeBr_6_ are more ductile, while others are more brittle.

## Conclusion

Here we have reported the photoelectric conversion properties and stability of 42 inorganic double perovskite materials based on density functional theory. Starting from the construction of double perovskite crystal structures, the data mining algorithm in informatics is introduced into high-throughput computing data analysis. First of all we discuss the valence band maximum (VBM) and conduction band minimum (CBM) of 42 compounds, and compare the band gaps of two efficient organic/inorganic perovskite materials. Among them, there are 39 kinds of crystals with their tolerance factors which are between 0.8 and 1.10. Then, the important parameters such as dielectric function, PDOS curve, elastic modulus, shear modulus and Poisson's ratio of the crystals are analyzed. Moreover, we investigate the maximum value of the valence band of these materials and the size of the valence band of the electron transport layer material, hole transport layer and back electrode material. These results are helpful to analyze the efficiency of carrier migration of these materials. We further screen out 14 kinds of crystalline perovskite crystals with energy band gaps between 1.33 eV and 2.40 eV. Then we analyze the minimum edge of the conduction band of these materials (Cs_2_ZrI_6_, Cs_2_HfI_6_, Cs_2_SeI_6_, Cs_2_TeI_6_, Rb_2_HfI_6_, Rb_2_SeI_6_, Rb_2_TeI_6_, Rb_2_GeCl_6_ Cs_2_SeBr_6_ , Rb_2_SeBr_6_ and Rb_2_HfBr_6_) is larger than − 4.6 eV, and the maximum value of the valence band lower than − 5.4 eV, refer to Fig. 7. Considering the above factors, we have reduced the range of crystals screened.

From the absorption spectrum, the light absorption curves of Cs_2_SeI_6_, Cs_2_SeBr_6_ Rb_2_SeI_6_, Rb_2_SeBr_6_ and Rb_2_SeCl_6_ fall in a relatively large range in the visible light region. The conclusion obtained according to the energy band theory is in well agreement with the absorption spectrum. The stability factor should be further considered, and the formation energy is an important factor for the thermodynamic stability of the reaction. The results show that the formation of compounds containing Cs and Rb elements is roughly the same, and the perovskite crystals containing Cs are more stable. Among several positive tetravalent valence elements, the perovskite crystal structure containing Zr and Hf is more stable. However, its light absorption efficiency is not very high. Taking into consideration with its band gap size and light absorption properties Cs_2_SeI_6_, Cs_2_SeBr_6_ and Rb_2_SeBr_6_ can be used as ideal candidates for light absorption materials. This work can not only guide experiments and design experiments rationally, but also reduce research and development costs, shorten research time, and improve the success rate of material design. Above all, it can provide an important theoretical basis for the industrialization of perovskite solar cells.

## Data Availability

The datasets used and analysed during the current study available from the corresponding author on reasonable request.
